# Chinese herbal medicine for ulcerative colitis: product-level clinical evidence, mechanistic appraisal, and a roadmap to precision translation

**DOI:** 10.3389/fphar.2026.1976448

**Published:** 2026-10-08

**Authors:** Yingxiu Wu, Jianxiong Ren, Huibiao Li, Shiyu Qi, Wei Ke

**Affiliations:** 1 Foshan Hospital of Traditional Chinese Medicine, Foshan, Guangdong, China; 2 The Eighth Clinical Medical College of Guangzhou University of Chinese Medicine, Foshan, Guangdong, China; 3 Guangzhou University of Chinese Medicine, Guangzhou, China; 4 The First Affiliated Hospital of Guangzhou University of Chinese Medicine, Zhongshan Hospital, Zhongshan, Guangdong, China

**Keywords:** aryl hydrocarbon receptor, botanical drug, Chinese herbal medicine, gut microbiota, pharmacovigilance, precision medicine, product characterization, ulcerative colitis

## Abstract

Chinese herbal medicine (CHM) is widely used for ulcerative colitis (UC), but product heterogeneity, variable trial quality, and limited long-term safety data preclude class-level efficacy claims. This critical narrative review therefore evaluates evidence at the level of the defined botanical drug product, formulation, route, dose, and manufacturing process. The review focuses on traditional formulas, processed botanical drugs, and proprietary preparations with documented use in Chinese medical practice. Purified plant metabolites such as curcumin and common flavonoids are not treated as uniquely Chinese interventions; they are discussed only when they are part of a defined CHM-related product or when pivotal natural-product trials provide a relevant product-level comparator. Primary randomized trials show heterogeneous results: higher-dose curcumin trials and the curcumin-QingDai combination provide signals of benefit, low-dose curcumin was negative, and indigo naturalis shows dose-related efficacy accompanied by important safety concerns. Mechanistically, the strongest evidence comes from human target engagement or causal perturbation, exemplified by mucosal CYP1A1 induction with curcumin-QingDai and loss of indigo naturalis efficacy in Ahr-deficient mice. Most other mechanistic claims remain preclinical and model-dependent. Translation requires taxonomically authenticated and chemically characterized products, measured exposure, causal mechanism testing, objective CORE-IBD/STRIDE-II-aligned outcomes, CONSORT-CHM reporting, and prospective, batch-traceable pharmacovigilance. No molecular, microbial, or traditional-pattern biomarker has yet shown a prospectively validated treatment-by-biomarker interaction for a defined CHM product. Precision translation should therefore be regarded as a prospective research program rather than an achieved clinical state.

## 1 Introduction

Ulcerative colitis (UC) is a chronic, immune-mediated disease of the colonic mucosa characterized by relapsing bleeding, urgency, diarrhea, fatigue, and impaired quality of life. Contemporary care is no longer judged solely by short-term symptomatic improvement. Guideline-directed targets now include corticosteroid-free clinical remission, endoscopic improvement or remission, normalization or substantial reduction of inflammatory biomarkers, durable maintenance, and prevention of hospitalization, colectomy, and treatment-related harm. The therapeutic landscape has expanded from 5-aminosalicylates (5-ASA agents) and corticosteroids to anti-tumor necrosis factor agents, anti-integrin and anti-interleukin biologics, Janus kinase inhibitors, and sphingosine-1-phosphate receptor modulators. Living guidelines and comparative evidence syntheses have accordingly raised the evidentiary standard against which any complementary intervention should be assessed (Le Berre et al., 2023; Ananthakrishnan et al., 2024; Singh et al., 2024b; Singh et al., 2024b; Rubin et al., 2025).

This progress has not eliminated unmet need. A substantial proportion of patients do not respond to an initially selected advanced therapy, lose response over time, or discontinue treatment because of infection, thromboembolic or cardiovascular risk, intolerance, cost, access, or preference. Acute severe UC continues to require rapid escalation and sometimes colectomy, while population-level studies document persistent hospitalization and surgical burden (Buie et al., 2023; Dai et al., 2023; Singh A. et al., 2024). The resulting therapeutic question is not whether Chinese herbal medicine (CHM) can be described as anti-inflammatory, but whether a reproducible herbal product adds clinically meaningful benefit within a treat-to-target pathway without delaying effective rescue therapy.

In this review, CHM is used as a historical field label but the unit of inference is a defined botanical drug product used within Chinese medical practice: a classical formula, a processed botanical drug, or a proprietary preparation with a traceable composition, route, and manufacturing description. Purified plant metabolites are not classified as Chinese by chemical identity. Curcumin, quercetin, baicalin, berberine, and related metabolites occur across botanical sources and pharmacological traditions; when they are discussed here, the text distinguishes a source-linked CHM mechanism from a general natural-product observation. Curcumin trials are considered contextual comparators because they are pivotal to the UC evidence base and because curcumin is incorporated into the CurQD product, but Western-manufactured curcumin formulations are not treated as proof of CHM efficacy. Accordingly, Zataria multiflora and a European anthocyanin-rich extract are not included in the clinical evidence table because they are not Chinese botanical medicines.

The clinical literature is similarly heterogeneous. A systematic review of randomized trials identified signals for several botanical drug interventions in active UC, but the certainty of evidence was commonly low or very low because of small trials, variable formulations, incomplete reporting, and inconsistent outcomes (Iyengar et al., 2024). An outcomes-reporting review further showed that CHM trials often used non-standard composite response measures rather than internationally interpretable clinical, endoscopic, histological, and biomarker endpoints (Zhang X. et al., 2024). Formula-level network meta-analysis can generate hypotheses about comparative performance, but indirect ranking cannot compensate for weak randomization, product heterogeneity, or lack of objective central reading (Zeng Y. et al., 2024; Zhao et al., 2024).

The clinical evidence is interpreted below by product rather than by molecule name or therapeutic tradition. This analysis separates non-unique natural-product comparators from defined Chinese botanical preparations, examines pivotal primary trials individually, and treats missing manufacturing information as an evidentiary limitation. This product-level approach is necessary because a favorable result with one dose, formulation, or route cannot be generalized to products that merely share a plant metabolite or traditional label.

This review advances three linked propositions. First, the evidentiary unit is the defined and reproducibly manufactured product and route, not the name of a broadly distributed plant metabolite. Second, mechanistic confidence should rise from association to measured exposure, human target engagement, and causal perturbation rather than from the number of enriched pathways. Third, efficacy and long-term safety must be evaluated together within contemporary UC treat-to-target care. Pharmacovigilance is therefore part of the evidence chain rather than a post-marketing afterthought, because small induction trials cannot characterize rare, delayed, or batch-specific harms (Figure 1).

**FIGURE 1:**
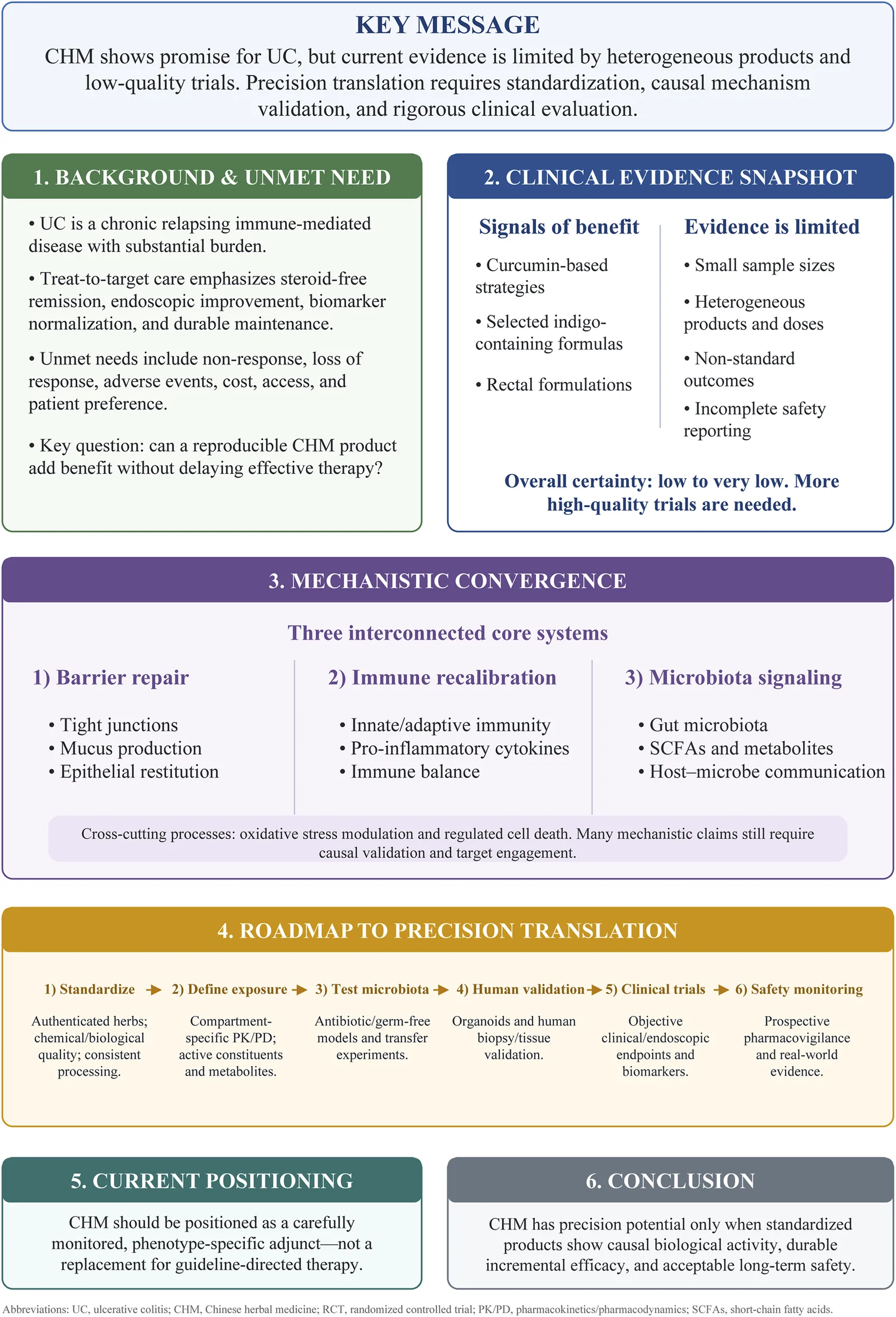
Product-level clinical evidence and precision-translation framework for Chinese herbal medicine in ulcerative colitis.

## 2 Narrative review methods, scope, and evidence appraisal

### 2.1 Review design, search, and selection

This article is a critical narrative review rather than a systematic review or meta-analysis. The final literature search was conducted on 16 September 2026. PubMed/MEDLINE was searched directly from database inception without a date restriction, using the complete search strings reproduced in Supplementary Methods S1; targeted publisher searches and backward/forward citation tracing were then used to verify pivotal trials, product descriptions, safety reports, mechanistic experiments, consensus standards, and 2026 publications. No language filter was applied to the PubMed queries, although full-text appraisal was predominantly based on English-language reports. CNKI, Wanfang, VIP, and SinoMed were not systematically searched in the present narrative review. This creates a risk of English-language indexing and selective-publication bias that is especially important in a review of Chinese botanical drug products. Because this limitation may introduce selection bias, Chinese-language coverage was cross-checked against prior evidence syntheses that searched Chinese databases; such cross-checking does not substitute for a systematic search of Chinese-language databases.

Eligible clinical evidence comprised randomized or controlled studies in adults with UC in which the investigated product, route, dose, comparator, and treatment period could be identified. Mechanistic evidence was included when it involved a defined botanical drug product or a source-linked plant metabolite and contributed to exposure, target engagement, causal perturbation, epithelial repair, immune regulation, or microbiota-mediated mechanisms. Exclusion criteria comprised non-UC studies, interventions without a defensible link to Chinese medical practice, non-primary protocols for the efficacy table, purely *in silico* studies for the representative mechanistic tables, and duplicate publications. Potential duplicate Chinese- and English-language reports were matched by author group, center, enrollment period, sample size, intervention, trial registration, and outcome definitions; when reports represented the same cohort, the most complete primary report was used for efficacy inference. Screening was not performed independently in duplicate, and a prospective screening log was not maintained. Accordingly, no screened-record denominator is reported. The evidence audit comprises eight pivotal primary randomized clinical trials in Table 1, six representative product/formula mechanistic entries in Table 2, and six source-linked plant metabolite entries in Table 3; detailed search documentation is provided in the Supplementary Material.

**TABLE 1:** Pivotal product-level randomized clinical trials in ulcerative colitis, with RoB 2 and GRADE appraisal.

Intervention/setting	Design and sample	Product, dose, comparator	Principal result	RoB 2	GRADE	Product-level interpretation
Curcumin - contextual natural-product comparator (not CHM-specific)	multicenter DB RCT; n = 89 quiescent UC (Hanai et al., 2006)	Curcumin 2 g/day + sulfasalazine/mesalamine vs. placebo; 6 months, then 6-month follow-up	Relapse among protocol completers 2/43 (4.65%) vs. 8/39 (20.51%); ITT also significant (P = 0.049)	Some concerns	Low	Maintenance signal for this dose/formulation; not evidence for CHM or all curcumin products
Curcumin - contextual comparator	DB RCT; n = 50 active mild-moderate UC (Lang et al., 2015)	Curcumin 3 g/day + mesalamine vs. placebo + mesalamine; 4 weeks	Clinical remission 53.8% vs. 0%; response 65.3% vs. 12.5%; endoscopic remission 38% vs. 0%	Some concerns	Low	Positive high-dose induction signal; small sample and formulation-specific
Curcumin - contextual comparator	DB RCT; n = 62 active mild-moderate UC (Kedia et al., 2017)	Curcumin 450 mg/day + mesalamine 2.4 g/day vs. placebo; 8 weeks	No significant remission benefit: 31.3% vs. 27.3%; 21/62 did not complete	High	Very low	Negative low-dose study; demonstrates dose/exposure dependence and attrition risk
Curcumin-QingDai (CurQD) - defined hybrid product	DB placebo RCT; 42 randomized, mITT n = 41 (Ben-Horin et al., 2024)	CurQD 3 g/day (1.5 g curcumin +1.5 g Qing-Dai) vs. placebo; 8 weeks	Week-8 co-primary response 43% vs. 8%; remission 50% vs. 8%; endoscopic improvement 75% vs. 20%; mucosal CYP1A1 increased	Some concerns	Low	Product-specific efficacy plus human AHR target engagement; confirmatory trial needed
Indigo naturalis (Qing-Dai)	multicenter DB dose-ranging RCT; n = 86 (Naganuma et al., 2018)	0.5, 1.0, or 2.0 g/day vs. placebo; 8 weeks	Clinical response 69.6%, 75.0%, 81.0% vs. 13.6%; trial stopped after external PAH safety signal	Some concerns	Low	Clear short-term efficacy signal but unresolved product identity and delayed safety constrain routine use
HMPL-004 (Andrographis paniculata extract)	multicenter DB RCT; n = 224 (Sandborn et al., 2013)	Standardized leaf extract 1,200 or 1,800 mg/day vs. placebo; 8 weeks	Clinical response 60% with 1,800 mg/day vs. 40% placebo; 1,200 mg/day not significant; remission not significantly different	Some concerns	Moderate	Relatively well-characterized botanical-drug trial; supports only HMPL-004, not a botanical class effect
Xilei San suppository	DB placebo RCT; n = 30 refractory ulcerative proctitis (Fukunaga et al., 2012)	Traditional multi-material suppository 0.1 g/day vs. placebo; day 14 and relapse through day 180	Relapse-free at day 180: 81.8% vs. 16.7%; clinical/endoscopic/histologic improvements reported	Some concerns	Low	Route-specific small trial; preparation includes botanical, mineral, and animal-derived materials
Qing-Chang-Hua-Shi (QCHS) granules	multicenter DB placebo RCT; n = 119 (Shen et al., 2021)	QCHS 125 g/day + 5-ASA 4 g/day vs. placebo + 5-ASA; 12 weeks	Clinical remission 31.48% vs. 12.50%; response 92.59% vs. 72.92%; 102/119 completed	Some concerns	Low	Defined 11-botanical formula with adjunctive signal; incomplete source-level chemistry/batch reporting limits transferability

**TABLE 2:** Representative product/formula mechanistic evidence with explicit translational-level assignment.

Level/product	Level/product	Level justification	Main limitation/next test
Level 2 - Curcumin-QingDai	Human RCT with mucosal CYP1A1 pharmacodynamic readout	Clinical efficacy accompanied by human mucosal evidence of AHR-pathway engagement	Small product-specific trial; quantify exposure, replicate target engagement and efficacy prospectively (Ben-Horin et al., 2024).
Level 3 - Indigo naturalis	Ahr-deficient mouse perturbation	Benefit and IL-10/IL-22 responses were abrogated in Ahr-deficient mice, supporting causal target dependence	Preclinical model and variable processed product; connect source species/chemistry/exposure to human mucosa (Kawai et al., 2017).
Level 3 - Osbeckia opipara	AHR/IL-22/STAT3 perturbation in experimental colitis	AHR inhibition abolished reported therapeutic effects, providing pathway-dependence evidence	Single preclinical program; replicate and establish human exposure (Su et al., 2026).
Level 4 - Shaoyao decoction	Animal/cellular immune-barrier and microbiota studies	Reproducible phenotypes are reported, but causal mediation and transferability remain incomplete	Product chemistry, measured exposure, and microbiota/receptor perturbation (Wu et al., 2023; Zhang et al., 2024c).
Level 3 - Huangqin decoction	Microbiota-tryptophan-AHR-stem-cell experiments	Antibiotic/AHR perturbation in the published 2026 study moves the claim beyond descriptive omics	Replicate across batches/models and verify human mucosal exposure/target engagement (Li et al., 2026a).
Level 4 - Wumei Wan	Goblet-cell differentiation/mucus-barrier experiments	Lineage-specific barrier hypothesis with chemical fingerprinting, but direct target dependence is not yet established	Replicate batches; test TGF-beta/Smad necessity and human organoid responses (Feng et al., 2026).

**TABLE 3:** Focused, level-graded appraisal of source-linked plant metabolites used as mechanistic probes in CHM-related research; these metabolites are not unique to Chinese medicine.

Level/plant metabolite and linked source	Evidence and level justification	Scope limitation
Level 3 - indigo/indirubin; processed indigo naturalis	Ahr-deficient experiments support causal dependence for indigo-related AHR signaling (Kawai et al., 2017).	Not unique to a single source species; infer only to a characterized indigo naturalis product/batch
Level 3 - paeoniflorin; Paeonia lactiflora-derived botanical drugs	Microbial indole-3-lactate/autophagy work includes microbiota-dependence tests, supporting causal microbial involvement (Fan et al., 2020; Ma et al., 2023).	Defined metabolite is not unique to a formula; clinical exposure remains unresolved
Level 3–2′-hydroxycinnamaldehyde; Cinnamomum-derived context	Direct STAT3 targeting with genetic/pharmacological perturbation constrains the mechanism (Chen et al., 2024).	Natural-product mechanism, not evidence for all Cinnamomum-containing CHM products
Level 4 - berberine/palmatine; Coptis chinensis-related context	Macrophage/metabolic and barrier effects are reported with defined alkaloids; the evidence remains predominantly preclinical (Chen et al., 2024).	Need metabolite-specific human exposure, selectivity, and explicit product linkage
Level 4 - baicalin/baicalein; Scutellaria baicalensis-related context	AHR/IL-22, T-cell, microbiota, and formulation studies provide a coherent but mainly preclinical hypothesis (Li et al., 2020; Yang et al., 2024a; Huang et al., 2025).	Do not extrapolate from a purified metabolite to the source botanical drug or formula
Level 4 - ginsenosides; Panax ginseng-related context	Congener-specific barrier, mitophagy, and inflammasome phenotypes are reported (Liu et al., 2018; Zhou et al., 2024a).	Congeners are distinct metabolites; require congener-specific pharmacokinetics and target dependence

### 2.2 Translational hierarchy, clinical bias assessment, and reporting standards

An author-developed five-level translational-maturity framework is used to organize mechanistic and product-level evidence. Level 1 denotes replicated clinical efficacy with objective endpoints and adequate product definition; Level 2 denotes clinical efficacy accompanied by human pharmacodynamic or tissue target-engagement evidence; Level 3 denotes preclinical causal perturbation, such as genetic deletion, selective inhibition, rescue, antibiotic depletion, germ-free transfer, fecal microbiota transplantation, or metabolite replacement; Level 4 denotes descriptive animal or cellular evidence without decisive causal perturbation; and Level 5 denotes *in silico* network pharmacology, docking, or pathway enrichment without direct experimental validation. This hierarchy is a translational heuristic, not a validated certainty-of-evidence system and not a substitute for GRADE (Balshem et al., 2011). Every entry in Tables 2, 3 now carries an explicit level and a brief justification.

For the clinical trials in Table 1, risk of bias was assessed using the Cochrane RoB 2 framework for the principal efficacy result, and certainty was summarized using GRADE concepts. Because most products are represented by a single pivotal trial, the GRADE label is applied to the current product-specific body of evidence for the stated primary efficacy outcome, not to CHM as a class. Domain-level RoB 2 judgments and downgrading reasons are provided in Supplementary Table S3. Trial endpoints were interpreted against CORE-IBD and STRIDE-II, which emphasize patient-reported outcomes, objective endoscopic assessment, biomarkers, safety, and durable treat-to-target outcomes (Turner et al., 2021; Ma et al., 2022).

Product reporting was audited against the Frontiers Four Pillars of Best Practice and the ConPhyMP framework for botanical extract characterization (Heinrich et al., 2022). For each clinically tested intervention, the audit extracted, when available, botanical identity, composition and ratios, processing/extraction, formulation and route, manufacturer/batch information, quantitative chemistry, contaminant testing, dose, comparator, background therapy, sample size, endpoint definition, duration, and follow-up. Missing source information is recorded as not reported (NR) rather than inferred. Botanical names were reconciled against Plants of the World Online and/or MPNS on 16 September 2026; accepted names, authorities, and families for retained multi-botanical preparations and source-linked plant taxa are provided in Supplementary Table S2; when a primary source does not support species-level attribution, this is recorded as NR rather than inferred. A completed ConPhyMP Supplementary Tables S1, S2A checklist accompanies this article as Supplementary Material.

## 3 Clinical evidence and positioning in contemporary UC care

### 3.1 Induction of remission: What is clinically credible?

Curcumin requires a scope distinction. Curcumin is a widely distributed plant metabolite and is not intrinsically a Chinese herbal medicine. Accordingly, the curcumin trials are treated as natural-product comparators rather than class-level CHM evidence. Their inconsistency is nevertheless instructive. In maintenance UC, Hanai et al. randomized 89 patients in remission to curcumin 2 g/day or placebo in addition to sulfasalazine or mesalamine for 6 months; relapse among protocol completers was 2/43 (4.65%) versus 8/39 (20.51%), and the intention-to-treat analysis also favored curcumin (P = 0.049) (Hanai et al., 2006). In active mild-to-moderate UC, Lang et al. randomized 50 patients to curcumin 3 g/day or placebo added to mesalamine for 4 weeks; clinical remission was 53.8% versus 0%, clinical response 65.3% versus 12.5%, and endoscopic remission 38% versus 0% (Lang et al., 2015). By contrast, in the 2017 negative low-dose trial, Kedia et al. randomized 62 patients to curcumin 450 mg/day or placebo with mesalamine for 8 weeks and found no significant difference in clinical remission (31.3% vs. 27.3%), response, or mucosal healing; 21 participants did not complete the study (Kedia et al., 2017). These trials argue against treating the name “curcumin” as a sufficient product definition: dose, formulation, exposure, background therapy, and outcome definition materially change the inference.

The curcumin-QingDai combination (CurQD) is best considered a defined hybrid product rather than a classical formula. In the randomized, double-blind, placebo-controlled phase, 42 patients were randomized and 41 were included in the prespecified modified intention-to-treat analysis (28 CurQD and 13 placebo). At week 8, the co-primary clinical-plus-objective response occurred in 43% versus 8% (P = 0.033); clinical response in 85.7% versus 30.7% (P < 0.001); clinical remission in 50% versus 8% (P = 0.01); and endoscopic improvement in 75% versus 20% (P = 0.036). A ≥50% reduction in fecal calprotectin occurred in 46.4% versus 15.4% (P = 0.08) (Ben-Horin et al., 2024). Importantly, CurQD uniquely increased mucosal CYP1A1 expression, providing human evidence of aryl hydrocarbon receptor (AHR) pathway engagement. The small trial supports confirmatory development of this specific product; it does not establish efficacy for curcumin, QingDai, or CHM as classes.

Indigo naturalis (Qing-Dai) illustrates why efficacy and delayed toxicity must be evaluated together. In the multicenter dose-ranging trial, 86 patients with active UC were randomized to 0.5, 1.0, or 2.0 g/day indigo naturalis or placebo for 8 weeks. Clinical response was 13.6% with placebo and 69.6%, 75.0%, and 81.0% across the three indigo groups; the trial was terminated after an external case of pulmonary arterial hypertension (PAH) in a patient who had self-purchased indigo naturalis for 6 months, although no serious adverse event occurred within the randomized trial itself (Naganuma et al., 2018). A later Japanese nationwide survey identified 877 users among 49,320 patients with UC and reported 107 adverse events in 91 users (10.4%), including liver dysfunction in 40 (4.6%), PAH in 11 (1.25%), and intussusception in 10 (1.14%), four of whom required surgery (Naganuma et al., 2019). More recent long-term follow-up of 20 Qing-Dai-associated PAH cases found a median exposure of 4.8 years before diagnosis (range 0.5–15 years); despite improvement after withdrawal and PAH therapy, 35% had persistent PAH (Isobe et al., 2025). Short induction-trial tolerability therefore cannot exclude delayed vascular toxicity after prolonged exposure.

Three additional primary trials deserve product-level discussion. HMPL-004, a standardized Andrographis paniculata (Burm.f.) Nees leaf extract, was evaluated in 224 patients with active UC; clinical response at week 8 was 60% with 1,800 mg/day versus 40% with placebo, whereas the 1,200 mg/day group did not differ significantly from placebo, and remission differences were not statistically significant (Sandborn et al., 2013). Xilei San, a traditional multi-material suppository that contains processed indigo naturalis together with non-botanical mineral and animal-derived materials, was tested in 30 patients with refractory ulcerative proctitis; 81.8% of the active-treatment group remained relapse-free at day 180 versus 16.7% with placebo (Fukunaga et al., 2012). Qing-Chang-Hua-Shi (QCHS) granules were evaluated in 119 patients with moderately active, mesalazine-refractory UC receiving background 5-ASA 4 g/day; week-12 clinical remission was 31.48% with QCHS versus 12.50% with placebo and clinical response was 92.59% versus 72.92% (Shen et al., 2021). These studies are clinically informative only at the level of the investigated product and route. Their product-characterization completeness is summarized in Supplementary Table S1, and missing manufacturing or chemistry information is treated as a translational limitation rather than silently filled in.

### 3.2 Maintenance, steroid-sparing, and advanced-therapy settings

Maintenance is a distinct indication that cannot be inferred from short induction trials. The Hanai curcumin study provides a proof-of-concept comparator for maintenance design, but curcumin is not classified here as CHM-specific and the trial does not establish a class effect. Future maintenance studies of defined CHM products should enroll patients in objectively verified remission, standardize concomitant therapy, and assess sustained corticosteroid-free clinical and endoscopic remission, biomarkers, treatment escalation, hospitalization, colectomy, and cumulative toxicity over at least 6–12 months. Chronic exposure increases the importance of batch stability and delayed safety surveillance.

The role of CHM alongside biologics and small molecules is almost unexplored. Modern comparative analyses show that advanced therapies have meaningful but heterogeneous efficacy, onset, and safety profiles (Ananthakrishnan et al., 2024; Singh et al., 2024b). A CHM add-on trial in this setting should therefore target a specific unmet need—partial response, residual urgency despite biomarker improvement, steroid tapering, or prevention of secondary loss of response—rather than simply compare a formula plus usual care with usual care in an unselected population. Pharmacokinetic interactions and overlapping immunosuppression must be prespecified, especially with Janus kinase inhibitors, calcineurin inhibitors, and thiopurines. The randomized TACOS study and histological analyses of tofacitinib demonstrate the speed and mechanistic depth now expected in severe and moderate-to-severe UC trials (Singh et al., 2024a; van Gennep et al., 2024).

Rectal delivery deserves separate development because it can maximize colonic exposure while limiting systemic exposure in proctitis and left-sided disease. Suppository and enema formulations are common in Chinese practice, and recent preclinical work on Qingchang suppository suggests local modulation of Th1/Th17 responses (Cao et al., 2025). Nevertheless, a rectal botanical drug product requires the same controls as an oral drug: uniform dissolution, mucosal tolerability, microbiological quality, retention feasibility, and dose–exposure characterization. It should be compared with optimized rectal 5-ASA, not with an undertreated control.

The most defensible present-day positioning is product-specific and adjunctive. A standardized product may be studied as an add-on to optimized 5-ASA in a defined mild-to-moderate population; a steroid-sparing program should target corticosteroid-free remission; and a maintenance program must demonstrate both relapse prevention and long-term safety. Products evaluated after advanced-therapy failure should be studied only within protocols that prevent delay of rescue treatment. None of these positions implies interchangeability with guideline-recommended therapy. Table 1 therefore lists primary controlled trials individually and adds product-specific RoB 2 and GRADE judgments; detailed product characterization is provided in Supplementary Table S1.

### 3.3 Outcome standardization and interpretation

Clinical outcome selection should be anchored to contemporary IBD standards rather than to non-validated “total effective rate” composites. CORE-IBD identifies patient-reported symptoms, quality of life, endoscopy, biomarkers, and safety as core domains for randomized IBD trials, while STRIDE-II distinguishes short-, intermediate-, and long-term treat-to-target goals, including symptomatic response/remission, normalization of C-reactive protein and fecal calprotectin, and endoscopic healing (Turner et al., 2021; Ma et al., 2022). CHM trials should therefore prespecify validated clinical response/remission, endoscopic improvement/remission, histological activity where relevant, fecal calprotectin, corticosteroid-free remission, sustained response, treatment escalation, hospitalization, and quality of life. Central endoscopic and histological reading is especially important in small trials.

Traditional syndrome classification can be retained as a prespecified stratification variable, but any predictive claim requires a prospectively specified treatment-by-pattern interaction and independent replication. Separate within-subgroup P values are not evidence of effect modification. Molecular, microbial, cytokine, or metabolite measures should likewise be treated as pharmacodynamic or exploratory mediators unless prospectively linked to a product-specific treatment interaction and objective clinical benefit.

Safety reporting must include denominators, exposure time, severity, seriousness, causality, withdrawals, laboratory abnormalities, and events of special interest. “No obvious adverse reactions” is not an adequate safety result. Trials of products containing indigo naturalis, anthraquinone-rich botanical drugs, aristolochic-acid-risk species, other potentially hepatotoxic botanical drugs, or potent cytochrome P450 (CYP) enzyme or P-glycoprotein modulators require targeted surveillance. Reproductive status, pregnancy, infection risk, anticoagulant use, and concomitant immunosuppression should be explicitly recorded.

## 4 Mechanistic evidence: from association to causal dependence

### 4.1 Epithelial, mucus, and regenerative barriers

The intestinal barrier is a dynamic tissue system rather than a static layer of tight-junction proteins. It comprises epithelial lineage allocation, mucus production and glycosylation, antimicrobial defense, junctional permeability, epithelial metabolism, stem-cell renewal, stromal support, and coordinated restitution after injury. In UC, inflammatory cytokines, neutrophil oxidants, microbial products, and epithelial cell death disrupt this system. Contemporary studies implicate cytokine-driven junctional damage, myeloperoxidase-mediated chlorination, altered mucin O-glycosylation, and impaired stem-cell signaling (Kaminsky et al., 2021; Cartwright et al., 2024; Ou et al., 2024; Wang T. et al., 2024). Therefore, an intervention should not be described as “repairing the barrier” solely because ZO-1 or occludin expression increased after inflammation decreased.

Barrier-oriented CHM studies are abundant but highly heterogeneous. To avoid implying that all named formulas are compositionally equivalent or equally validated, the synthesis focuses on products for which botanical composition can be documented and for which the experiment contributes a distinct mechanistic step. Huangqin decoction provides a microbiota-AHR-stem-cell example; Wumei Wan provides a lineage-specific mucus-barrier hypothesis; and Osbeckia opipara provides perturbation-based evidence along the AHR/IL-22/STAT3 axis (Feng et al., 2026; Li R. et al., 2026; Su et al., 2026). These examples support biological plausibility, but none permits a class-level statement that CHM “repairs the barrier.” Botanical definitions for the multi-botanical preparations and source-linked plant taxa included in the mechanistic appraisal are provided in Supplementary Table S2; unresolved source-species attribution is explicitly marked NR.

Defined plant metabolites can be useful mechanistic probes, but they should not be treated as ethnopharmacologically unique. Paeoniflorin, baicalin/baicalein, ginsenosides, matrine/oxymatrine, berberine/palmatine, and related metabolites are discussed only when linked to a specified source botanical drug or product. Their evidentiary value depends on measured exposure and perturbation, not on presumed membership in a Chinese-medicine chemical class. Recent Osbeckia opipara work combined chemical characterization with AHR-pathway perturbation and provides a more constrained preclinical example, although it remains model-dependent (Su et al., 2026).

Mucus biology is an underdeveloped translational endpoint. Goblet-cell differentiation, MUC2 secretion, O-glycosylation, and microbial access to the epithelium are central to colonic homeostasis (Yao et al., 2021; Wei et al., 2023; Gustafsson and Hansson, 2025). ADORA3-mediated ketogenesis and IL-22–dependent occludin regulation illustrate how metabolism and immune signaling intersect with epithelial differentiation and junctional repair (Nong et al., 2024; Zeng X. et al., 2024). Current mucosal-healing and Chinese-medicine barrier reviews also support moving from single-marker assays toward integrated functional repair endpoints (Zong et al., 2023; Zhou et al., 2024b; Mansouri et al., 2025). Future CHM studies should measure mucus thickness, glycan structure, epithelial lineage composition, and functional permeability rather than relying on a small panel of immunoblots. Secretory epithelial subsets should be treated as active regulators of inflammation and repair rather than passive structural cells (Huang et al., 2026). Wumei Wan has been associated with TGF-β/Smad-dependent goblet-cell differentiation and restoration of the colonic mucus barrier, providing a testable lineage-specific hypothesis (Feng et al., 2026).

Regenerative claims also require temporal resolution. A treatment that prevents injury induced by dextran sulfate sodium (DSS) before exposure is not equivalent to one that restores established ulceration. Therapeutic designs should begin after endoscopic or histological injury is present, compare repair kinetics, and examine whether epithelial recovery persists after drug withdrawal. Human colonoids and biopsy-derived organoids can test epithelial-autonomous effects, while co-culture with immune, stromal, and microbial components can expose context dependence.

A cross-cutting limitation is dependence on chemically induced colitis models. DSS begins with epithelial and mucus injury followed by secondary inflammation and does not reproduce the multifactorial, relapsing immune-mediated initiation of human UC. Disease severity also varies with DSS molecular characteristics and dose, mouse strain, sex, housing, diet, and baseline microbiota. A benefit observed in one preventive DSS experiment should therefore be interpreted as biological plausibility rather than as a prediction of clinical efficacy. Stronger programs should test treatment after established disease, use complementary models, reproduce findings across batches and laboratories, and incorporate human organoid, biopsy, or *ex vivo* systems.

### 4.2 Innate and adaptive immune recalibration

UC involves coordinated dysfunction of epithelial sensing, neutrophils, macrophages, dendritic cells, innate lymphoid cells, and adaptive lymphocytes. The therapeutic objective is not global immunosuppression but restoration of a mucosal response that controls microbes while permitting repair. Recent syntheses highlight interleukin networks and JAK/STAT signaling as central nodes (Nakase et al., 2022; Aebisher et al., 2024; Long et al., 2025). The clinical effectiveness of targeted JAK inhibition and its histological effects provide a useful benchmark for claims that plant metabolites regulate the same pathway (Singh et al., 2024a; van Gennep et al., 2024).

At the innate level, studies of Chinese botanical drug products frequently report inhibition of TLR–MyD88–NF-κB signaling, NLRP3 activation, macrophage M1 polarization, neutrophil recruitment, or pyroptosis. Gegen Qinlian decoction has been associated with effects on T-cell balance, NLRP3, and the microbiota; oxymatrine was associated with NLRP3-mediated pyroptosis; salidroside with macrophage pyroptosis; and arbutin with neutrophil extracellular traps and microbial remodeling (Liu X. et al., 2023; Hu et al., 2024; Qin et al., 2024; Sun et al., 2024). Selective pharmacological or genetic perturbation is required to determine whether these nodes mediate efficacy or simply reflect reduced tissue injury. Neutrophil biology is bidirectional, with antimicrobial, injurious, and reparative states that cannot be inferred from cell counts alone (Yu et al., 2025). Monomethylsulochrin provides an example of a defined candidate linked to IL-17/TNF modulation and microbial changes, but its proposed dual mechanism remains preclinical (Xing et al., 2026).

Macrophage polarization is often described using an oversimplified M1/M2 binary. Palmatine and a hepatic oxidative metabolite have been linked to AMPK/NF-κB signaling and macrophage phenotype, while Th1-derived signals can promote barrier-damaging macrophage programs (Ruan et al., 2023; Yang T. et al., 2024; Huang et al., 2025). IL-17 can also generate atypical macrophage states rather than fitting a binary model (Nishikawa et al., 2014). Single-cell and spatial approaches should therefore characterize macrophage states, localization, and ligand–receptor interactions instead of reporting only CD86 and CD206. Wenyang decoction has likewise been connected to macrophage polarization and the PI3K/AKT/mTOR/HIF-1α axis (Li S. et al., 2026). Epigenetic regulation of mucosal innate immunity may further shape these states, but DNA-methylation signatures require cell-specific and longitudinal validation before they can function as treatment biomarkers (Zhang et al., 2026).

Adaptive studies commonly report changes in Th17/Treg or Th1/Th2 balance after formula or plant metabolite exposure. These observations can be secondary to reduced antigen load or epithelial injury and should not be interpreted as target-level mechanisms from cell frequencies alone. Stronger evidence requires lineage-specific perturbation, receptor dependence, adoptive transfer, or *ex vivo* responses of patient-derived cells at measured concentrations. Source-linked examples in Tables 2 and 3 are graded according to whether such causal steps were performed.

Two AHR studies illustrate the distinction between association, human target engagement, and causal target dependence. In the CurQD trial, mucosal CYP1A1 - a canonical AHR-response gene - was uniquely upregulated after treatment and not in placebo-, mesalamine-, or biologic-treated comparison samples, providing Level 2 human pharmacodynamic target-engagement evidence (Ben-Horin et al., 2024). In the experimental indigo naturalis study, improvement in DSS colitis and induction of IL-10/IL-22 responses were abrogated in Ahr-deficient mice, providing Level 3 evidence that AHR signaling was required for the observed effect (Kawai et al., 2017). These examples are stronger than a post-treatment increase in pathway proteins because the first measures a pathway response in human mucosa and the second removes the proposed target genetically. They do not, however, erase product-specific exposure and safety constraints.

JAK/STAT-related claims require similar discipline. Experimental inhibition of TLR4/JAK2/STAT3 and direct targeting of STAT3 by 2′-hydroxycinnamaldehyde provide examples of pathway-constrained studies (Chen et al., 2024; Huang et al., 2024). IL-6/STAT3 can affect epithelial proliferation and damage, and mTOR/HIF-1 signaling can amplify inflammation (Li et al., 2021; Wang et al., 2024e). Because STAT phosphorylation is a common downstream response, its normalization alone does not identify a primary drug target. Direct binding, selectivity profiling, and rescue with constitutively active pathway components would substantially raise confidence. MAPK-oriented natural-product evidence is useful for organizing candidate mechanisms, but pathway normalization should remain subordinate to exposure, selectivity, and perturbation-based validation (Li et al., 2025).

### 4.3 The microbiota–metabolite–immune axis

Microbiota modulation is frequently invoked in CHM research and is especially vulnerable to overinterpretation. UC-associated communities vary with geography, diet, medication, inflammation, sample type, sequencing method, and baseline host biology (Shen et al., 2018; Franzosa et al., 2019; Bajaj et al., 2024). A rise in a taxon labelled as beneficial does not demonstrate mediation, and relative-abundance shifts can result from changes elsewhere in the community. Stool findings also may not represent mucosa-associated communities. The appraisal therefore assigns higher evidence levels only to experiments that test microbial necessity, transferability, receptor dependence, or metabolite sufficiency.

Representative product-linked studies connect Chinese formulas or defined botanical drugs to microbial functions, but the evidence tables emphasize mechanistically informative findings rather than cataloguing every reported taxonomic shift. Recent Huangqin decoction studies link microbial tryptophan metabolism, AHR signaling, and epithelial differentiation; Shaoyao decoction provides a microbiota-immune example; Shaoyao decoction is retained as a microbiota-immune example; and polysaccharide studies are discussed only when product identity and causal microbial tests are adequate. Descriptive single-source abundance changes are not used to support a causal mechanism.

Short-chain fatty acids (SCFAs) are recurrent candidate mediators. Astragalus polysaccharides were reported to restore SCFA production and Th17/Treg homeostasis in a microbiota-dependent manner; Codonopsis polysaccharide was linked to an SCFA/G-protein-coupled receptor/NLRP3 pathway; and sea buckthorn berries have been linked to butyrate biosynthesis associated with Faecalibaculum rodentium (Song et al., 2025; Zhang et al., 2025; Zhou et al., 2025). Independent literature supports immunoregulatory effects of butyrate and other SCFAs on epithelial and T-cell function (Zhou et al., 2018; Akhtar et al., 2022; Hodgkinson et al., 2023; Yang W. et al., 2024). Broader synthesis of butyrate biology emphasizes that concentration, site of production, and host context determine whether an observed association is therapeutically relevant (Kalkan et al., 2025). Causality is strongest when antibiotic depletion removes efficacy, transfer reproduces efficacy, and metabolite replacement restores it.

Tryptophan metabolism provides a testable host-microbe bridge. Paeoniflorin has been linked to microbial indole-3-lactate and epithelial autophagy, whereas the 2026 Huangqin decoction study linked microbial tryptophan metabolism to AHR activation and intestinal stem-cell differentiation (Fan et al., 2020; Li R. et al., 2026). Untargeted metabolomics alone is insufficient: targeted quantification, isotope tracing where feasible, microbial depletion or transfer, receptor antagonism, and compartment-specific exposure are needed to distinguish mediation from correlation. Accordingly, the evidence level assigned to the 2026 Huangqin study in Table 2 reflects the perturbation actually performed rather than the breadth of the omics association.

Bile acids constitute a third candidate module. Baicalin magnesium was reported to act through a microbiota–bile-acid axis, while gallic acid influenced bile-acid metabolism and ILC3-related responses (Leng et al., 2024; Zhang L. et al., 2024). Bile-acid metabolites can directly shape Th17 and Treg differentiation (Hang et al., 2019). Experiments with taurohyodeoxycholic acid provide an additional comparator linking bile-acid signaling with Th1/Th2 and Th17/Treg balance (Lv et al., 2023). Future studies should distinguish primary from secondary bile acids, quantify conjugated species, measure receptor activation at FXR and TGR5, and test whether microbial bile-acid transformation the same need to distinguish the pharmacology of a defined extract or plant metabolite from broad claims about the source botanical drug (Ding et al., 2026).

The fungal and viral components of the microbiome are largely neglected. Patient data suggest that fungal dysbiosis and Dectin-1–related cytokine signaling may contribute to UC (Azizollah et al., 2024). Stable commensal communities may also provoke different epithelial responses among patients (Inciuraite et al., 2024). Multi-kingdom sequencing, strain-resolved metagenomics, metatranscriptomics, and spatial sampling should be integrated with medication and diet records. Work on mucosal immunity, pathogenic adherent-invasive *Escherichia coli* (AIEC) programs, and microbiota-dependent B-cell repertoires further illustrates that taxonomic shifts can have context-specific immune consequences (Leccese et al., 2024; Sengupta and Sen, 2024; Wang J. et al., 2024). Without these controls, a generic statement that CHM “regulates the gut microbiota” is scientifically weak.

Microbiome causality requires more than a change in relative abundance. Consistent with the recent consensus on causality and preclinical models in microbiome research, stronger evidence should integrate complementary systems - including antibiotic depletion where appropriate, gnotobiotic or human-microbiota-associated animals, genetically modified hosts, organoids or organ-on-chip models - and should account for donor, diet, housing, and baseline-community variation (Metwaly et al., 2025). For CHM-related studies, a practical sequence is association, necessity, transferability, and sufficiency: reproducible community/metabolite change; loss of efficacy after depletion or pathway blockade; transfer of part of the phenotype by microbiota or a defined consortium; and reproduction of the key effect by a defined strain, metabolite, or receptor agonist. Table 2 assigns higher translational levels only when such perturbation is present.

### 4.4 Immunometabolism, oxidative stress, and regulated cell death

Oxidative stress is both a driver and a consequence of mucosal inflammation. Neutrophil oxidants, epithelial mitochondrial dysfunction, hypoxia, and altered nutrient metabolism can amplify barrier failure and cytokine release. Sinomenine has been associated with Nrf2/HO-1 signaling, while matrine and related interventions have been linked to oxidative-stress control and microbial remodeling (Mao et al., 2024; Niu et al., 2024). These pathways are biologically plausible, but generic reductions in malondialdehyde or increases in antioxidant enzymes do not identify a primary mechanism.

Regulated cell-death programs provide a more specific lens. Pyroptosis and NLRP3 activation are targeted by oxymatrine and salidroside-related studies (Liu X. et al., 2023; Sun et al., 2024). Ferroptosis has emerged as a proposed CHM-responsive pathway, with oxymatrine and palmatine studies reporting effects on lipid-peroxidation and iron-dependent injury (Gao et al., 2024; Ji et al., 2024; Wang et al., 2024d). Necroptosis of epithelial cells has been linked to P2Y14R–PKA/CREB/RIPK1 signaling (Liu et al., 2024; Wang K. et al., 2024). Excessive apoptosis, endoplasmic-reticulum stress, autophagy, and cytokine-induced epithelial death also intersect in UC (Woznicki et al., 2021; Wan et al., 2022; Tran et al., 2023).

The translational problem is that markers of several death programs often change simultaneously in injured tissue. To claim ferroptosis, studies should demonstrate iron dependence, lipid-peroxide accumulation, characteristic rescue by selective inhibitors, and exclusion of alternative death pathways. To claim pyroptosis, cleavage and pore formation should be connected to inflammasome dependence and cell-specific localization. For autophagy, static LC3 measurements are inadequate without flux assays. These standards matter because pathway labels can otherwise become interchangeable narratives attached to the same histological improvement.

Metabolic reprogramming also links immune and epithelial states. AHR agonism, glycolysis–lactate–STAT3 signaling, PPARγ/AMPK pathways, and mitochondrial quality control have each been implicated in experimental colitis (Xu et al., 2017; Liu et al., 2018; Lv et al., 2018; Liu S. et al., 2023). Ginsenoside Rd has been connected to p62-driven mitophagy and NLRP3 inactivation (Liu et al., 2018). Such work suggests that natural products may change the energetic constraints under which immune cells and epithelium respond. Human validation will require metabolic flux, cell-type resolution, and exposure–response analyses rather than static endpoint metabolomics.

### 4.5 Stromal, vascular, and neuroimmune dimensions

Mucosal healing depends on subepithelial stromal cells, endothelial integrity, enteric glia, nerves, and immune–stromal communication. Recent work describes stromal support of epithelial homeostasis and shows that myeloid IL-1 signaling can suppress fibroblast-derived neuregulin-1 during early repair (Ayansola et al., 2024; Qiu et al., 2024). Vascular adhesion and chemokine programs contribute to leukocyte recruitment, and herbal flavonoid preparations have been studied in endothelial contexts (Elia and Guglielmi, 2018; Xue et al., 2023). These compartments are rarely included in CHM studies, creating a major blind spot.

Neuroimmune effects are similarly plausible but underdeveloped. Berberine has been reported to modulate interactions among enteric glia, epithelial cells, and immune cells, while cholinergic manipulation can alter experimental colitis and dysbiosis (Li et al., 2020; Singh et al., 2020). Gut-derived metabolites also affect distant neural and microglial states (Deng et al., 2024). It would be premature to label these findings a proven gut–brain mechanism of CHM in UC, but they justify studies of visceral sensitivity, urgency, autonomic function, and patient-reported symptoms alongside mucosal endpoints.

Broad surveys of plant-derived metabolites may be useful for hypothesis generation, but the evidence tables are intentionally restricted to mechanistically informative examples. Table 2 contains only representative defined products or formulas and assigns an explicit translational level with a justification. Table 3 contains only source-linked plant metabolites used as mechanistic probes; it is not a general UC-biology inventory and does not imply that any listed metabolite is unique to Chinese medicine. Non-CHM comparators such as air-pollution epigenetics, cAMP signaling, NOD2 biology, olsalazine, walnut oil, and MCC950 are not included in Table 3 and are discussed, where relevant, only as contextual biology in the text.

## 5 Causal pharmacology, precision treatment, and safety

### 5.1 Botanical identity and reproducible product definition

A named formula is not yet a reproducible investigational product. Product definition in this section follows the Frontiers Four Pillars of Best Practice and the ConPhyMP framework (Heinrich et al., 2022). In a narrative review, ConPhyMP functions as a source-reporting audit rather than as a manufacturing checklist for a newly produced extract: product-level information is transcribed from the primary studies, and missing information is recorded as not reported (NR) rather than reconstructed. Product definition should include accepted botanical names with authorities and families, medicinal parts, taxonomic authentication and vouchers where applicable, processing, extraction solvent and yield, excipients, manufacturer and batch, storage and stability, contaminant testing, and fit-for-purpose chemical characterization. For extracts, marker selection and chromatographic or spectrometric fingerprinting should be justified by identity, exposure, or pharmacological relevance. The completed ConPhyMP Supplementary Tables S1, S2A checklist accompanies this article as Supplementary Material, while Supplementary Tables S1, S2 document which clinically tested products meet or fail these reporting elements.

Quality markers should be selected because they support identity, exposure, stability, or pharmacological relevance rather than merely because they are analytically convenient. A chromatographic fingerprint can demonstrate similarity but cannot by itself establish pharmacological equivalence. For indigo naturalis, source species and processing are particularly important because the processed drug can be produced from indigo-yielding species including Strobilanthes cusia (Nees) Kuntze [syn. Baphicacanthus cusia (Nees) Bremek.; Acanthaceae], Persicaria tinctoria (Aiton) Spach [syn. Polygonum tinctorium Aiton; Polygonaceae], and Isatis tinctoria L [including the synonym Isatis indigotica Fortune ex Lindl.; Brassicaceae]. Traditional processing involves soaking/fermentation of stems and leaves, removal of plant material, addition of lime under alkaline conditions, oxidation/precipitation of indigo-related metabolites, refining, and drying. When a clinical publication did not identify the source species, processing batch, or quantitative chemistry, that information is recorded as NR rather than assuming pharmacopoeial equivalence. An HPLC fingerprint analysis of 11 Osbeckia opipara batches identified 13 shared chromatographic peaks and high inter-batch similarity, illustrating how batch characterization can support identity while not establishing therapeutic equivalence by itself (Su et al., 2024).

Formulation can materially change exposure and efficacy. Curcumin is the clearest example: the positive 2–3 g/day trials and the negative 450 mg/day study do not support a formulation-independent class effect. The same principle applies to poorly soluble plant metabolites and extract products. A trial of one formulation should not be cited as proof for all products sharing a metabolite name; comparative dissolution, chemical profile, and exposure data are needed when manufacturing changes.

### 5.2 Pharmacokinetics, microbial biotransformation, and target engagement

Oral botanical-drug products create at least three pharmacological compartments: the upper gastrointestinal lumen, systemic circulation, and colonic lumen/mucosa. Low systemic bioavailability does not imply inactivity if local colonic concentrations are high, but local activity must be measured rather than assumed. Parent plant metabolites can be transformed by host enzymes or microbes into products with different potency and targets. Studies should quantify relevant parent and biotransformation products in plasma, stool, and colonic tissue over time and compare those concentrations with the concentrations used in mechanistic assays.

Direct target engagement remains uncommon. Appropriate methods include chemical proteomics, cellular thermal shift assays, activity-based probes, competition experiments, binding assays, and target-mutant rescue where chemically feasible. The direct STAT3 study of 2′-hydroxycinnamaldehyde illustrates a constrained target claim, whereas network pharmacology remains hypothesis-generating until selectivity, exposure, and perturbation are demonstrated (Chen et al., 2024). For complex formulas, chemical fingerprints and spectrum-effect analyses can prioritize fractions, but active metabolites and binding partners still require orthogonal confirmation.

Combination pharmacology should distinguish additivity, synergy, antagonism, and exposure interaction. A multi-herb formula may increase solubility, alter metabolism, or reduce toxicity, but such claims require factorial or response-surface designs. Omics can reveal coordinated responses, as shown in work applying multi-omic approaches to tanshinone IIA (Zhu et al., 2022). Omics alone cannot identify the causal ingredient or target; it should be coupled to fractionation, reconstitution, and perturbation. Multi-omic analysis of Qinlian Yuyang decoction similarly provides a discovery framework only when the resulting networks are followed by component-resolved validation, measured exposure, and independent replication (Cao et al., 2026).

### 5.3 Precision translation and biomarker-guided selection

No proposed CHM precision biomarker - molecular, microbial, pharmacodynamic, or traditional-pattern-based has yet demonstrated a prospectively validated treatment-by-biomarker interaction for a defined product in an adequately powered independent clinical trial. The precision-translation framing in this review is therefore prospective, not a description of an achieved standard of care. Biomarker analyses should be prespecified, interaction-based, externally replicated, and linked to a product-specific mechanism and measured exposure. Traditional syndrome classification can be retained as a stratification variable, but it should not substitute for biomedical disease activity measures or be declared predictive on the basis of separate within-subgroup P values.

Future precision trials should prespecify the biomarker, assay, cut-point, expected direction of interaction, statistical interaction test, and replication plan. An AHR-oriented product could, for example, prospectively test a baseline mucosal AHR-response signature; a fermentable polysaccharide product could test a defined microbial functional capacity. These are hypotheses for trial design, not validated prescribing rules. A recent Mendelian-randomization analysis of preferences for polyphenol-rich plant foods provides etiological context for plant-derived metabolites and IBD, but it does not constitute treatment-efficacy or predictive-biomarker evidence for a defined CHM product (Yue et al., 2026).

Traditional pattern information may be retained when diagnostic criteria and assessor training are prespecified. It should be analyzed alongside objective UC activity and should be called predictive only if a treatment-by-pattern interaction is demonstrated prospectively and replicated. At present, no such validated product-specific interaction has been established.

### 5.4 Safety, pharmacovigilance, interactions, and regulatory pathways

Safety is product-, dose-, route-, duration-, and patient-specific. The perception that “natural” implies safe is particularly hazardous in UC, where patients may be immunosuppressed, anemic, malnourished, pregnant, anticoagulated, or at risk of urgent surgery. Potential harms include intrinsic hepatotoxicity or nephrotoxicity, pulmonary vascular effects, ischemic injury, electrolyte disturbance, infection, allergy, reproductive toxicity, and contamination with heavy metals, pesticides, mycotoxins, microbes, or undeclared pharmaceuticals. Species substitution and processing errors can introduce additional risk.

Botanical drug-conventional drug interactions are plausible through CYP enzymes, UDP-glucuronosyltransferases, P-glycoprotein and other transporters, protein binding, coagulation, and overlapping immune effects. The clinically relevant question is not whether an extract changes an enzyme *in vitro*, but whether a standardized clinical dose changes exposure to or toxicity of mesalazine, corticosteroids, thiopurines, calcineurin inhibitors, JAK inhibitors, anticoagulants, or other concomitant medicines. Interaction studies should prioritize high-risk combinations and human-relevant concentrations.

Pharmacovigilance should combine active trial surveillance, laboratory monitoring, standardized causality assessment, and longitudinal registries that record product name, manufacturer, batch or lot, route, dose, exposure dates, and concomitant medicines. The nationwide indigo naturalis survey provides an interpretable denominator: 91 of 877 users (10.4%) experienced 107 reported adverse events, including liver dysfunction in 40, PAH in 11, and intussusception in 10 (Naganuma et al., 2019). Spontaneous-reporting databases may add signal-detection value but should not be used to estimate incidence without a reliable exposure denominator or product identity. The 2025 long-term PAH series further shows why short-term tolerability is insufficient: PAH was diagnosed after a median 4.8 years of Qing-Dai exposure, and persistent disease remained in a substantial minority despite withdrawal and treatment (Isobe et al., 2025).

Regulatory translation is jurisdiction-specific. In China, the National Medical Products Administration (NMPA) drug-registration framework and the 2023 Special Provisions for Traditional Chinese Medicines Registration place safety, efficacy, quality, and manufacturing consistency within the registration pathway for traditional Chinese medicines (National Medical Products Administration, 2023). In the United States, a botanical product intended to treat UC enters the investigational new drug and new drug application pathway; FDA botanical-drug guidance emphasizes control of raw materials, manufacturing consistency, and the totality of chemical, biological, and clinical evidence (Food and Drug Administration, 2016). In the European Union, herbal medicinal products may follow traditional-use registration, well-established-use, or full marketing-authorization routes; traditional-use registration is a regulatory route and should not be interpreted as proof of efficacy for UC (European Medicines Agency, 2026). These pathways affect development cost, release testing, pharmacokinetics, multicenter trial requirements, reimbursement, distribution, and access. Cost-effectiveness and equitable access become meaningful only after reproducible efficacy and manufacturing consistency have been established. Product-level safety, pharmacovigilance, and regulatory risks are summarized in Table 4.

**TABLE 4:** Consolidated safety, pharmacovigilance, and regulatory risk matrix for product development in UC.

Risk source	Potential consequence	Surveillance	Development/regulatory control
Indigo naturalis/Qing-Dai	Liver dysfunction, PAH, intussusception; delayed vascular toxicity	Baseline cardiopulmonary/hepatic assessment as indicated; symptom-triggered testing; longitudinal follow-up	Nationwide survey: 91/877 users with AEs; PAH 11/877; long-term PAH series median exposure 4.8 years. Define source species, batch, dose, duration, stopping rules
Anthraquinone-rich botanical drugs	Diarrhea, electrolyte disturbance, melanosis, potential hepatic toxicity	Electrolytes, liver tests, stool pattern, exposure duration	Quantify relevant metabolites; control processing and maximum duration
Alkaloid-rich products	CYP/transporter interactions and metabolite-specific toxicity	Medication reconciliation; targeted interaction studies	Measure parent and biotransformation products at clinical exposure
Concomitant immunosuppressants/antithrombotics	Additive infection, cytopenia, hepatic, bleeding or thrombotic risk	CBC/liver tests when indicated; infection screening; event adjudication	Predefine high-risk combinations and exposure-safety analyses
Manufacturing/identity failure	Batch heterogeneity, substitution, contaminants, undeclared pharmaceuticals	Validated release testing; supplier audit; batch traceability	ConPhyMP/Four Pillars product definition; retained trial batches and certificates where available
Long-term maintenance exposure	Cumulative or delayed toxicity and rare events	Registry follow-up; prespecified events of special interest	Batch-level pharmacovigilance; exposure time and denominator-based reporting

## 6 Translational roadmap and research priorities

A translational roadmap for Chinese herbal medicine (CHM) in ulcerative colitis can be organized as a sequence of eight evidence gates: (1) botanical identity and product chemistry; (2) exposure and biotransformation; (3) target engagement and causal mechanism; (4) model validity and microbiome causality; (5) randomized clinical proof-of-concept; (6) objective treat-to-target efficacy; (7) confirmatory reporting and prospectively tested precision interactions; and (8) long-term safety, regulation, implementation, cost, and access. The framework presented here is intended as a research and development heuristic rather than an adopted regulatory or industry standard. Its purpose is to ensure that increasingly complex mechanistic or clinical claims are supported by a continuous evidence chain, because uncertainty in product identity, exposure, or causal mechanism cannot be compensated for by downstream omics complexity or nominal clinical significance.

Clinical development should begin with a target-product profile that defines the investigational product, intended UC population, position in therapy, route, dosing schedule, expected onset and duration of effect, clinically meaningful incremental benefit, key safety liabilities, and prespecified go/no-go criteria. Product identity and characterization should satisfy ConPhyMP and the Frontiers Four Pillars recommendations; microbiome-dependent claims should meet contemporary causality criteria; efficacy should be evaluated against disease-specific treat-to-target outcomes; and long-term development should incorporate pharmacovigilance, regulatory feasibility, manufacturing scalability, and access. This sequence places product reproducibility and biological plausibility before claims of comparative or precision benefit.

Mechanism-embedded randomized controlled trials offer an efficient route for linking exposure to biological response and clinical outcome. Blood, stool, and mucosal biopsies should be collected at prespecified time points to quantify relevant plant metabolites and biotransformation products, assess target engagement, characterize microbial genes or metabolites when mechanistically justified, and measure epithelial or immune pharmacodynamics alongside objective disease activity. Multi-omics analyses are most informative when they test a prespecified causal chain; exploratory networks generated after the clinical outcome is known should be treated as hypothesis-generating rather than confirmatory.

Clinical efficacy and reporting should follow standards appropriate to both UC and complex botanical interventions. CORE-IBD and STRIDE-II provide a disease-specific basis for judging symptoms, biomarkers, endoscopic activity, safety, and durability, whereas the CONSORT Extension for Chinese Herbal Medicine Formulas provides intervention-specific guidance for prospective registration, randomization, allocation concealment, placebo construction, full formula and processing disclosure, prespecified statistical analysis, intention-to-treat reporting, missing-data sensitivity analyses, and transparent harms reporting (Cheng et al., 2017). Trial batches should be retained and analytically archived whenever feasible. Claims of precision treatment require more than subgroup response: they require a prespecified treatment-by-biomarker interaction and independent replication.

Preclinical development should move beyond reliance on preventive DSS experiments. Therapeutic DSS designs, oxazolone and TNBS models, genetically susceptible models, human organoids, biopsy explants, immune-epithelial co-cultures, and microbiota-transfer systems address different elements of UC biology and should be selected according to the causal question being tested. Positive findings should be reproduced across laboratories and product batches, and dose selection should be linked to clinically plausible exposure rather than arbitrary multiples of crude product mass. Sex, age, diet, housing, and baseline microbiota should be reported because each can materially alter experimental phenotypes. For microbiome-mediated claims, combinatorial model systems and explicit consideration of donor, recipient, and model limitations are particularly important (Metwaly et al., 2025).

Comparative-effectiveness and implementation research become informative only after reproducible efficacy and manufacturing consistency have been demonstrated. At that stage, clinically relevant questions include whether a validated CHM product reduces corticosteroid exposure, improves adherence or patient-reported outcomes, affects work productivity or treatment cost, alters sequencing with advanced therapies, or introduces a monitoring burden that changes its net value. These outcomes are important to patients and health systems, but they complement rather than replace objective control of intestinal inflammation (Constantin et al., 2019).

Progress across these evidence domains would be facilitated by coordinated multicenter collaboration. A shared research infrastructure could define a core outcome set, minimum botanical and chemical reporting requirements, a harmonized adverse-event lexicon, reference analytical methods, and biobanks linking retained product batches with blood, stool, and mucosal specimens. Such collaboration should include gastroenterologists, pharmacognosists, analytical chemists, trialists, statisticians, microbiome scientists, toxicologists, regulators, and patient partners, because product identity, mechanism, efficacy, and long-term safety cannot be established by any single discipline in isolation (Table 5).

**TABLE 5:** Proposed evidence gates for precision translation of CHM in UC.

Evidence gate	Minimum evidence	No-go signal
1. Botanical identity and product chemistry	ConPhyMP/Four Pillars: accepted taxonomy, medicinal part, processing, manufacturer/batch, contaminants, stability, quantitative fingerprint/markers	Identity, batch release, or stability cannot be demonstrated
2. Exposure and biotransformation	Plasma, stool and/or mucosal concentrations of parent and relevant biotransformation products; formulation-dependent bioavailability	Proposed active exposure is not reached in the relevant compartment
3. Target engagement and causal mechanism	Human target engagement where feasible; genetic/pharmacological perturbation or rescue at measured exposure	Only network enrichment, docking, or nonspecific protein-expression changes
4. Model and microbiome causality	Therapeutic designs, complementary models, human-derived systems; microbiome necessity/transfer/sufficiency tests consistent with consensus guidance	Benefit restricted to one preventive DSS experiment or descriptive microbiome shifts
5. Randomized clinical proof-of-concept	Defined product; credible randomized comparator; objective clinical/endoscopic endpoint and prespecified estimand	Only non-standard effective-rate or unblinded subjective improvement
6. Objective treat-to-target efficacy	CORE-IBD/STRIDE-II-aligned symptoms, biomarkers, endoscopy, safety, durability; adequate power and central reading where appropriate	No objective inflammation endpoint or clinically interpretable treatment effect
7. Confirmatory reporting and precision interaction	CONSORT-CHM reporting, prospective registration/SAP, complete product disclosure, ITT and missing-data analysis; prespecified treatment-by-biomarker interaction and external replication for precision claims	Post hoc subgroup without interaction test; formula/manufacturing drift; incomplete reporting
8. Long-term safety, regulation, implementation, cost and access	Exposure-aware pharmacovigilance, interaction studies, regulatory pathway, scalable GMP manufacture, comparative effectiveness, affordability/access	Commercial uptake precedes reproducible efficacy, manufacturing consistency, or acceptable monitoring/regulatory plan

## 7 Conclusion

Chinese herbal medicine for UC should be judged at the level of the defined product, not by the cultural label of a molecule or by a list of pathways. The clinical literature includes positive and negative trials, and the curcumin dose/formulation contrast, CurQD target-engagement data, indigo naturalis efficacy-safety tension, HMPL-004, Xilei San, and QCHS trials collectively show why formulation, route, exposure, background therapy, and manufacturing determine what can be inferred. Common plant metabolites such as curcumin and flavonoids are not unique to Chinese medicine and should not be presented as such. Mechanistically, the most persuasive evidence comes from human target engagement or causal perturbation; descriptive pathway or microbiome changes remain hypothesis-generating. At present, defined CHM products are most appropriately evaluated as adjunctive candidates and should not replace guideline-directed therapy without comparative evidence. A precision role remains prospective because no product-specific molecular, microbial, or traditional-pattern biomarker has yet shown a prospectively validated treatment-by-biomarker interaction. Progress depends on ConPhyMP-level product characterization, causal pharmacology, CORE-IBD/STRIDE-II-aligned outcomes, CONSORT-CHM reporting, long-term pharmacovigilance, and regulatory and access planning.

The evidence hierarchy should be interpreted conservatively: Level 2 human target engagement and Level 3 causal perturbation are uncommon, while most product-mechanism claims remain Level 4 and require replication, measured exposure, and human validation. The roadmap is a proposed research and development heuristic rather than an approved regulatory standard.

Future work should prioritize a smaller number of well-defined products, complete botanical and chemical characterization, causal experiments matched to measured exposure, CONSORT-CHM-compliant clinical reporting with UC core outcomes, and batch-traceable pharmacovigilance. These elements are prerequisites for credible claims about precision use, regulatory translation, cost-effectiveness, or access.
