# Supplementary material for: Chinese herbal medicine for ulcerative colitis: product-level clinical evidence, mechanistic appraisal, and a roadmap to precision translation
Source: Front Pharmacol. 2026 Oct 8;17:1976448. doi: 10.3389/fphar.2026.1976448 (PMC13687833; doi:10.3389/fphar.2026.1976448)
Supplement: Supplementary file 1 [file Table1.docx]

**ConPhyMP Checklist - Completed Tables 1 and 2A**

Journal: Frontiers in Pharmacology - Ethnopharmacology

Date of enquiry/self-assessment: 16 September 2026

Manuscript: Chinese Herbal Medicine for Ulcerative Colitis: Product-Level Clinical Evidence, Mechanistic Appraisal, and a Roadmap to Precision Translation

Authors: Yingxiu Wu, Jianxiong Ren, Huibiao Li, Shiyu Qi, Wei Ke

Important scope note: This is a narrative review; the review authors did not prepare or analytically characterize a new extract. The official ConPhyMP items are therefore completed as a manuscript-level source-reporting audit. Items requiring new extract analysis are marked N/A for the review authors; Supplementary Tables S1-S2 report whether the corresponding information was available in each primary clinical source. Missing source information is recorded as NR and treated as a limitation.

**Table 1. ConPhyMP checklist of information for reporting plant material and its initial processing (all extract types A, B, C)**

| **SECTION/TOPIC** | **ITEM NO.** | **CHECKLIST ITEM** | **YES** | **NO** | **N/A** | **PAGE/SECTION, IF ANY** |
| --- | --- | --- | --- | --- | --- | --- |
| Title and abstract | 1 | A clear and concise title including an informative abstract and balanced summary. | X |  |  | Title and Abstract |
| Description of the botanical drug | 2 | Botanical or morphological authentication of the plant material and taxonomic authentication (desirable is a combination with DNA barcoding, e.g., PCR, RFLP, genome sequencing) and the information must be included in a separate section of Material and Methods, if applicable, combined with the information required under item 3. | X |  |  | Section 5.1; Supplementary Table S2. Primary-source gaps are marked NR. |
| Description of the extract and extraction process | 3 | A separate section in Material and Methods covers the relevant information on the material investigated, including the full species name(s), authorities and family, and on the processing and extraction of the crude drug including traditional processing of the material used medicinally (fumigation, steaming, roasting, cooking, frying, etc.). | X |  |  | Section 5.1; Supplementary Tables S1-S2. Review transcribes source data and does not infer missing information. |
| Documentation of the legal basis for collection and processing | 4 | Full compliance with the Nagoya protocol, CITES, and all associated treaties including phytosanitary regulations. |  |  | X | N/A: no field collection, importation, or new extraction was undertaken by the review authors. |
| Description of product characteristics, in case of a finished (commercial) product | 5 | Information on the characteristics of the commercial products including batch number and date of production/best by information and regulatory status. | X |  |  | Supplementary Table S1 audits manufacturer/batch/regulatory information when reported; missing fields are NR. |

**Table 2A. ConPhyMP checklist of items for conducting and reporting analytical methods - extract type A**

| **SECTION/TOPIC** | **ITEM NO.** | **CHECKLIST ITEM** | **YES** | **NO** | **N/A** | **PAGE/SECTION, IF ANY** |
| --- | --- | --- | --- | --- | --- | --- |
| Type of extract | 1 | A - Confirm that the species or botanical drug under investigation is covered in a monograph in one of the national or regional pharmacopoeias. |  |  | X | N/A at review-author level; monograph/pharmacopoeial status is reported only when available in the source publication. |
| Preferred/main methods for extract characterisation/chemical analysis | 2 | Compliance with pharmacopoeial standards: (a) description of active ingredients in the botanical drug (if known) or analytical marker compounds; (b) analysis as defined in the monograph if the extract has not been supplied with a certificate; (c) if purchased, manufacturer and certificate of analysis. Preferred characterisation includes (a) triple chemical fingerprinting methods, each with one or more detection parameters; and (b) quantification of at least two marker compounds unless not feasible, with justification. |  |  | X | No extract was generated by the review authors. Supplementary Table S1 audits manufacturer, certificate of analysis (CoA)/batch and quantitative chemistry in the primary trials; gaps are NR. |
| Alternative methods for extract characterisation/chemical analysis | 3 | (a) Single chemical fingerprinting method with at least three different detection parameters; the same principle applies when MS or NMR is coupled to chromatography. (b) Quantification of at least two marker compounds unless not feasible, with justification of marker choice. |  |  | X | N/A for new analysis; Section 5.1 recommends fit-for-purpose fingerprinting and multi-marker quantification for future product development. |
| Use of reference standards | 4 | (a) Direct overlay of the chromatogram of the sample with that of an officially specified reference standard, if applicable. (b) Chromatographic fingerprinting: direct overlay of the sample chromatogram with official reference standards of powdered plant material or dry extract. |  |  | X | N/A for review authors; primary-source analytical reporting is audited rather than recreated. |
| Comparison of different extracts/samples of the same plants | 5 | Direct comparison of the chromatographic/spectroscopic system and/or scoring system for similarity to be followed. |  |  | X | N/A for review authors; the manuscript explicitly states that a fingerprint alone does not prove pharmacological equivalence and calls for cross-batch comparison. |

Checklist source: Heinrich M, Jalil B, Abdel-Tawab M, Echeverria J, Kulić Ž, McGaw LJ, et al. Best Practice in the chemical characterisation of extracts used in pharmacological and toxicological research - The ConPhyMP - Guidelines. Front Pharmacol. 2022;13:953205. doi:10.3389/fphar.2022.953205. Official tool: Society for Medicinal Plant and Natural Product Research (GA), https://ga-online.org/best-practice/.
