# Supplementary material for: Chinese herbal medicine for ulcerative colitis: product-level clinical evidence, mechanistic appraisal, and a roadmap to precision translation
Source: Front Pharmacol. 2026 Oct 8;17:1976448. doi: 10.3389/fphar.2026.1976448 (PMC13687833; doi:10.3389/fphar.2026.1976448)
Supplement: Supplementary file 2 [file Table2.docx]

Chinese Herbal Medicine for Ulcerative Colitis: Product-Level Clinical Evidence, Mechanistic Appraisal, and a Roadmap to Precision Translation

**Yingxiu Wu^1,2*^, Jianxiong Ren^1,2^, Huibiao Li^3^, Shiyu Qi^4^, Wei Ke^1,2*^**

**Affiliation:**

1. Foshan Hospital of Traditional Chinese Medicine, Foshan, Guangdong 528000, China

2. The Eighth Clinical Medical College of Guangzhou University of Chinese Medicine, Foshan, Guangdong 528000, China

3. Guangzhou University of Chinese Medicine, Guangzhou, 510000, China.

4. The First Affiliated Hospital of Guangzhou University of Chinese Medicine, Zhongshan Hospital, Zhongshan, Guangdong, 528463, China.

***Correspondence:**

**Yingxiu Wu**

Foshan Hospital of Traditional Chinese Medicine, Foshan, Guangdong 528000, China; The Eighth Clinical Medical College of Guangzhou University of Chinese Medicine, Foshan, Guangdong 528000, China

Email: wyx20201206@163.com

**Wei Ke**

Foshan Hospital of Traditional Chinese Medicine, Foshan, Guangdong 528000, China; The Eighth Clinical Medical College of Guangzhou University of Chinese Medicine, Foshan, Guangdong 528000, China

Email: cosy_edu123@163.com

**Supplementary Methods S1. Search documentation**

**Supplementary Methods S1. Search documentation**

Review type: critical narrative review. Final search update: 16 September 2026. Directly searched database: PubMed/MEDLINE (database inception to final date; no date filter; no language filter). Targeted publisher searches and backward/forward citation tracing were used for source verification. CNKI, Wanfang, VIP and SinoMed were not systematically searched. This omission may bias the review toward English-indexed studies. Chinese-language coverage was therefore contextualized using prior systematic reviews that searched Chinese databases; this does not eliminate the selection-bias limitation.

**PubMed search A - clinical trials:**

("Colitis, Ulcerative"[Mesh] OR "ulcerative colitis"[tiab]) AND ("Drugs, Chinese Herbal"[Mesh] OR "Medicine, Chinese Traditional"[Mesh] OR "Chinese herbal medicine"[tiab] OR "Chinese herbal formula*"[tiab] OR "botanical drug*"[tiab] OR Qing-Dai[tiab] OR "indigo naturalis"[tiab] OR "Xilei San"[tiab] OR "Qing-Chang-Hua-Shi"[tiab] OR "Andrographis paniculata"[tiab] OR curcumin[tiab]) AND (random*[tiab] OR trial[tiab] OR placebo[tiab] OR "clinical trial"[pt])

**PubMed search B - translational/mechanistic evidence:**

("Colitis, Ulcerative"[Mesh] OR "ulcerative colitis"[tiab]) AND ("Chinese herbal medicine"[tiab] OR "Chinese herbal formula*"[tiab] OR Qing-Dai[tiab] OR "indigo naturalis"[tiab] OR "Huangqin decoction"[tiab] OR "Shaoyao decoction"[tiab] OR "Wumei Wan"[tiab] OR "Osbeckia opipara"[tiab]) AND (mechanis*[tiab] OR pharmacokinetic*[tiab] OR exposure[tiab] OR "target engagement"[tiab] OR microbiot*[tiab] OR metabolom*[tiab] OR AHR[tiab] OR "aryl hydrocarbon receptor"[tiab] OR barrier[tiab] OR organoid*[tiab] OR "fecal microbiota transplantation"[tiab] OR antibiotic*[tiab])

**PubMed search C - safety/pharmacovigilance:**

("ulcerative colitis"[tiab] OR "Colitis, Ulcerative"[Mesh]) AND (Qing-Dai[tiab] OR "indigo naturalis"[tiab] OR "Chinese herbal medicine"[tiab] OR "botanical drug"[tiab]) AND (safety[tiab] OR adverse[tiab] OR hepatotoxic*[tiab] OR "pulmonary arterial hypertension"[tiab] OR intussusception[tiab] OR pharmacovigilance[tiab])

**Selection and deduplication:**

Clinical inclusion required adult UC, an identifiable intervention/product and route, a controlled or randomized design, and clinically interpretable outcomes. Mechanistic inclusion required a defined product or source-linked plant metabolite and an experiment informative for exposure, target engagement, perturbation, barrier, immune, or microbiota mechanisms. Duplicate reports were matched by author group, institution, enrollment dates, sample size, intervention, registration number and outcomes; the most complete primary report was retained. Independent duplicate screening was not used. The narrative search did not prospectively record a screened-record denominator. Accordingly, no screened-record denominator is reported. The evidence audit comprises 8 pivotal primary clinical trials in Table 1, 6 mechanistic product/formula entries in Table 2, and 6 source-linked metabolite entries in Table 3.

**Search accounting and screening transparency**

| **Source** | **Search/screening method** | **Screened-record denominator** | **Evidence included in the product-level audit** |
| --- | --- | --- | --- |
| PubMed/MEDLINE | Direct narrative-review searches using the complete strings above; no date or language filter | Not prospectively logged; no screened-record denominator is reported | 8 pivotal primary clinical trials; mechanistic/safety references selected for the critical narrative argument |
| Publisher searches and backward/forward citation tracing | Targeted verification of pivotal trials, product details, consensus standards, safety reports, and 2026 publications | No formal denominator because these were verification/citation-chasing steps | Used to verify source-level data and publication status |
| CNKI, Wanfang, VIP, SinoMed | Not systematically searched | Not applicable | Potential Chinese-language/English-language indexing selection bias acknowledged in the manuscript; duplicate reports handled by cohort matching |

**Supplementary Table S1. Product-characterization audit of pivotal clinically tested interventions**

| **Study/product** | **Composition / source** | **Processing / extraction** | **Formulation / route** | **Manufacturer / batch** | **Quantitative chemistry** | **Contaminant testing** | **Dose** | **Comparator** | **Background therapy** | **Sample** | **Primary endpoint** | **Duration / follow-up** | **Audit note** |
| --- | --- | --- | --- | --- | --- | --- | --- | --- | --- | --- | --- | --- | --- |
| Hanai 2006 curcumin | Purified curcumin; botanical source NR | Purified product; extraction NR | Oral capsule | Manufacturer/batch NR | Quantitative purity/chemistry NR in primary report | Contaminants NR | 2 g/day | Placebo | Sulfasalazine/mesalamine | n=89 | Relapse during 6-month maintenance | 6 months + 6-month follow-up | Natural-product comparator; not CHM-specific |
| Lang 2015 curcumin | Purified curcumin; botanical source NR | Formulation details limited in report | Oral | Manufacturer/batch incompletely reported | Chemistry/purity incompletely reported | NR | 3 g/day | Placebo | Mesalamine | n=50 | Clinical remission at 4 weeks | 4 weeks | Comparator; small product-specific trial |
| Kedia 2017 curcumin | Purified curcumin; botanical source NR | 150-mg capsules; extraction NR | Oral | Source/manufacturer details limited | Purity/chemistry NR | NR | 450 mg/day | Placebo | Mesalamine 2.4 g/day | n=62 | Clinical remission at 8 weeks | 8 weeks | 21/62 did not complete; not CHM-specific |
| Ben-Horin 2024 CurQD | Curcumin + processed Qing-Dai; exact Qing-Dai source species NR in report | Defined combination; processed indigo naturalis material | Oral | Trial product defined; batch-level details incomplete for ConPhyMP audit | Curcumin purity and indigo/indirubin content were confirmed by LC-MS/MS; complete release specifications and batch profile were not fully reported | Contaminant testing NR | 3 g/day total (1.5 g curcumin + 1.5 g Qing-Dai) | Placebo | Stable conventional therapy per trial | 42 randomized; mITT 41 | Clinical response + objective response | 8-week induction; maintenance to week 16 | Human CYP1A1 target engagement; hybrid product |
| Naganuma 2018 indigo naturalis | Processed indigo naturalis; source species/batch not sufficiently specified for trial-level inference | Traditional processed drug; source-specific processing NR | Oral powder | Product/batch detail incomplete | Indigo/indirubin product chemistry insufficient for cross-batch equivalence | Contaminant testing NR | 0.5/1.0/2.0 g/day | Placebo | Background UC therapy controlled per protocol | n=86 | Clinical response at week 8 | 8 weeks; trial stopped early | Major product-characterization and delayed-safety concern |
| Sandborn 2013 HMPL-004 | Andrographis paniculata (Burm.f.) Nees [Acanthaceae], leaf extract | Ethanol/water 90:10 extraction; evaporation, spray-drying, homogenization/sieving | Oral 200-mg hard-gelatin capsules | Hutchison Medipharma; cGMP; controlled plantation; lot release described | Andrographolide marker; release specification ≥6%; trial lots approximately 8–10% | Release testing was reported; the complete contaminant panel was not fully specified in the primary report | 1,200 or 1,800 mg/day | Placebo | Conventional UC therapy per protocol | n=224 | Clinical response at week 8 | 8 weeks | Most reproducibly characterized product among pivotal trials |
| Fukunaga 2012 Xilei San | Multi-material traditional preparation: indigo naturalis plus calculus bovis, pearl powder, mirabilitum praeparatum, gypsum rubrum, borax, ammonium chloride, borneol | Traditional compounded preparation; detailed manufacturing/batch chemistry NR | Rectal suppository | Manufacturer/batch NR/incomplete | Quantitative chemistry NR | Contaminant testing NR | 0.1 g/day | Placebo suppository | Refractory to topical mesalamine/corticosteroid before enrollment | n=30 | Avoidance of relapse through day 180; early efficacy day 14 | 180 days | Not purely botanical; route-specific and incompletely characterized |
| Shen 2021 QCHS | 11-botanical formula; full composition in Supplementary Table S2 | Raw botanical drugs hot-water extracted, concentrated and dried; granules | Oral granules | Jiangyin Tianjiang Pharmaceutical (protocol); batch/certificate of analysis (CoA) detail incomplete | Complete quantitative multi-marker chemistry NR in clinical report | Contaminant testing NR | 125 g/day | Matched placebo | 5-ASA 4 g/day | n=119 | Clinical response/remission at week 12 | 12 weeks; protocol follow-up to 24 weeks | Formula defined, but ConPhyMP chemistry/batch data incomplete |

**Supplementary Table S2. Botanical nomenclature and composition audit for products/formulas included in the appraisal**

| **Product/formula** | **Type** | **Accepted botanical definition (authority; family; ratio when reported)** | **Processing / preparation** | **Interpretive note** |
| --- | --- | --- | --- | --- |
| Indigo naturalis (Qing-Dai) | Processed botanical drug; not a single species | Strobilanthes cusia (Nees) Kuntze [Acanthaceae] (syn. Baphicacanthus cusia (Nees) Bremek.); Persicaria tinctoria (Aiton) Spach [Polygonaceae] (syn. Polygonum tinctorium Aiton); Isatis tinctoria L. [Brassicaceae] (incl. synonym Isatis indigotica Fortune ex Lindl.) | Stems/leaves traditionally soaked/fermented; plant material removed; lime added under alkaline conditions; indigo-related metabolites oxidize/precipitate; refined and dried | Clinical trial source species frequently NR; do not assume interchangeability |
| HMPL-004 | Single-botanical standardized extract | Andrographis paniculata (Burm.f.) Nees [Acanthaceae], leaves | Controlled plantation; 90:10 ethanol/water extraction; spray-dried; andrographolide marker | Used as defined botanical drug comparator |
| Huangqin decoction | 4-botanical formula | Scutellaria baicalensis Georgi [Lamiaceae]; Paeonia lactiflora Pall. [Paeoniaceae]; Glycyrrhiza uralensis Fisch. ex DC. [Fabaceae]; Ziziphus jujuba Mill. [Rhamnaceae]; source study ratio 3:2:2:2 | Water decoction; two 30-min extractions; combined filtrates concentrated to 1 g/mL | Mechanistic exemplar in Table 2; composition and ratio transcribed from the 2026 source study |
| Shaoyao decoction | 9-botanical formula | Paeonia lactiflora Pall. [Paeoniaceae] 30 g; Angelica sinensis (Oliv.) Diels [Apiaceae] 15 g; Coptis chinensis Franch. [Ranunculaceae] 15 g; Areca catechu L. [Arecaceae] 6 g; Dolomiaea costus (Falc.) Kasana & A.K.Pandey [Asteraceae] 6 g (source report: Aucklandia costus/lappa); Rheum palmatum L./Rheum tanguticum Maxim. ex Balf./Rheum officinale Baill. [Polygonaceae] 9 g; Scutellaria baicalensis Georgi [Lamiaceae] 15 g; Cinnamomum cassia (L.) J.Presl [Lauraceae] 5 g; Glycyrrhiza uralensis Fisch. ex DC./G. inflata Batalin/G. glabra L. [Fabaceae] 6 g | Water decoction; repeated boiling and vacuum drying in source experiment | Source allows more complete formula definition; not a clinical product |
| Wumei Wan | 10-botanical formula | Prunus mume (Siebold) Siebold & Zucc. [Rosaceae]; Asarum heterotropoides F.Schmidt [Aristolochiaceae]; Zingiber officinale Roscoe [Zingiberaceae]; Coptis chinensis Franch. [Ranunculaceae]; Angelica sinensis (Oliv.) Diels [Apiaceae]; Aconitum carmichaelii Debeaux [Ranunculaceae]; Zanthoxylum bungeanum Maxim. [Rutaceae]; Cinnamomum cassia (L.) J.Presl / source-specific synonym usage [Lauraceae]; Panax ginseng C.A.Mey. [Araliaceae]; Phellodendron chinense C.K.Schneid. or source-specific P. amurense Rupr. [Rutaceae] | Traditional formula; source-study ratios and processing vary across reports, so product-level inference is study-specific | Barrier-focused mechanistic exemplar; product-level inference depends on the exact source-study botanical materials and processing |
| Osbeckia opipara | Single botanical | Osbeckia opipara C.Y.Wu & C.Chen [Melastomataceae] | Study-specific extract/fingerprint; see source report | Perturbation-based preclinical exemplar in Table 2 |
| Curcumin comparators (Hanai/Lang/Kedia) | Purified metabolite / non-CHM contextual comparator | Source botanical species not specified in pivotal clinical reports; therefore not assigned retrospectively | Commercial/purified curcumin formulations; source-processing details incompletely reported | Not classified as CHM-specific; missing botanical source is a product-characterization limitation, not filled by inference |
| Curcumin-QingDai (CurQD) | Defined hybrid product | Curcumin source botanical species NR in trial report; Qing-Dai source species NR in trial report. Potential indigo naturalis source taxa are listed separately in this audit. | 1.5 g/day curcumin + 1.5 g/day Qing-Dai dry powder in pH-dependent coated capsules; supplied by EviNature, manufactured at Bara Herbs GMP facility | Human CYP1A1 target engagement; source-species and full batch-chemistry gaps remain |
| Xilei San | Traditional mixed-origin suppository | Processed indigo naturalis is the botanical drug portion; source species NR in the clinical trial. Other listed materials include non-botanical mineral/animal-derived substances. | Rectal suppository; source-level processing and batch chemistry incompletely reported | Not a purely botanical formula; botanical identity inference limited to what the primary report provides |
| QCHS granules | 11-botanical formula | Primary clinical protocol reports materia-medica drug names and ratios: Coptidis Rhizoma 6 g; Scutellariae Radix 10 g; Patriniae Herba 15 g; Angelicae Sinensis Radix 10 g; Paeoniae Radix Alba 20 g; Angelicae Dahuricae Radix 12 g; Aucklandiae Radix 6 g; Sanguisorbae Radix 10 g; Lithospermi Radix 10 g; Rubiae Radix et Rhizoma 20 g; Glycyrrhizae Radix et Rhizoma 6 g. Where the clinical source does not state a unique source species, species is recorded as NR rather than inferred. | Raw botanical drugs were hot-water extracted, concentrated, dried, and formulated as granules; manufactured by Jiangyin Tianjiang Pharmaceutical Co., Ltd. in the trial protocol | Composition and ratios are reported, but complete species-level voucher/authentication and multi-marker chemistry are insufficient for full ConPhyMP reproducibility. Species are not assigned retrospectively where the clinical source does not identify a unique taxon. |
| Paeoniflorin source botanical context | Single source-linked plant taxon | Paeonia lactiflora Pall. [Paeoniaceae] | Source studies use purified paeoniflorin or related peony-derived preparations; product-specific processing varies by study. | Taxon verified for source-linkage only; purified paeoniflorin must not be treated as equivalent to a Paeonia botanical drug. |
| 2′-Hydroxycinnamaldehyde source context | Source-linked natural product | Cinnamomum-derived context; the cited mechanistic evidence does not justify retrospective assignment to a single source species (species NR for product-level inference). | Purified natural-product study; source-botanical processing is not the unit of inference. | No species-level CHM claim is made; this entry is used only as a causal-mechanism exemplar. |
| Berberine/palmatine source botanical context | Single source-linked plant taxon | Coptis chinensis Franch. [Ranunculaceae] | Purified alkaloid/metabolite studies; botanical drug extraction and processing are study-specific and not inferred. | Accepted taxon verified; evidence applies to the tested metabolite, not automatically to Coptis-containing formulas. |
| Baicalin/baicalein source botanical context | Single source-linked plant taxon | Scutellaria baicalensis Georgi [Lamiaceae] | Purified metabolite/formulation studies; extraction and formulation differ across experiments. | Accepted taxon verified; evidence applies to the tested metabolite/formulation rather than to all Scutellaria botanical drugs. |
| Ginsenoside source botanical context | Single source-linked plant taxon | Panax ginseng C.A.Mey. [Araliaceae] | Congener-specific purified-metabolite studies; source processing and formulation differ by experiment. | Accepted taxon verified; individual ginsenosides are distinct metabolites and cannot be generalized to ginseng products as a class. |

**Supplementary Table S3. RoB 2 domain judgments and GRADE rationale for Table 1 clinical entries**

| **Trial** | **D1 randomization** | **D2 deviations** | **D3 missing outcome data** | **D4 outcome measurement** | **D5 selective reporting** | **Overall RoB 2** | **GRADE** | **Main rationale** |
| --- | --- | --- | --- | --- | --- | --- | --- | --- |
| Hanai 2006 curcumin | Low | Low | Some concerns | Low | Some concerns | Some concerns | Low | Single modest-size study; imprecision and reporting limitations |
| Lang 2015 curcumin | Low | Low | Some concerns | Low | Some concerns | Some concerns | Low | Small sample; some objective outcomes available only in subsets; imprecision |
| Kedia 2017 curcumin | Low | Low | High | Low | Some concerns | High | Very low | 21/62 did not complete; serious attrition plus imprecision |
| Ben-Horin 2024 CurQD | Low | Low | Some concerns | Low | Some concerns | Some concerns | Low | Small single product-specific trial; mITT exclusion and imprecision |
| Naganuma 2018 indigo naturalis | Low | Low | Some concerns | Low | Some concerns | Some concerns | Low | Early termination for external safety signal; small dose groups; safety/precision limitations |
| Sandborn 2013 HMPL-004 | Low | Low | Some concerns | Low | Some concerns | Some concerns | Moderate | Largest pivotal trial and characterized product; single-study uncertainty remains |
| Fukunaga 2012 Xilei San | Some concerns | Low | Some concerns | Low | Some concerns | Some concerns | Low | Very small n=30; incomplete product reporting and imprecision |
| Shen 2021 QCHS | Low | Low | Some concerns | Low | Some concerns | Some concerns | Low | 17/119 did not complete; product chemistry incomplete; single-study imprecision |

Note: RoB 2 is outcome/result-specific. These judgments refer to the principal efficacy result summarized in Table 1. GRADE is ordinarily applied to a body of evidence; because most products here have one pivotal trial, the label summarizes the current product-specific body for that outcome and should not be interpreted as certainty for CHM as a class.

**Supplementary Table S4. Publication-status verification for 2026-dated references considered in the evidence appraisal**

Publication status was verified for 2026-dated references cited in the manuscript or considered in the evidence appraisal. Huangqin decoction is a published Chinese Medicine article with DOI 10.1186/s13020-025-01302-y. The Chinese herbal enema trial is an issue-year 2026 Journal of the Chinese Medical Association article that was electronically published on 19 June 2025 (DOI 10.1097/JCMA.0000000000001259). Its publication status is documented here, but it is not used as a pivotal product-level efficacy example in the focused synthesis.

| **Reference** | **Publication status checked 16 Sep 2026** | **DOI** |
| --- | --- | --- |
| Cao et al. 2026, Chin Med 21:100 | Published version of record | 10.1186/s13020-026-01380-6 |
| Chen et al. 2026, J Ethnopharmacol 364:121498 | Published version of record | 10.1016/j.jep.2026.121498 |
| Ding et al. 2026, World J Gastroenterol 32:114558 | Published version of record | 10.3748/wjg.v32.i1.114558 |
| Dong et al. 2026, Front Pharmacol 17:1738151 | Published version of record | 10.3389/fphar.2026.1738151 |
| Feng et al. 2026, J Ethnopharmacol 368:121837 | Published version of record | 10.1016/j.jep.2026.121837 |
| Gu et al. 2026, Drug Des Devel Ther 20:574142 | Published version of record | 10.2147/DDDT.S574142 |
| Huang et al. 2026, Curr Opin Gastroenterol 42:247-254 | Published version of record | 10.1097/MOG.0000000000001176 |
| Jiang et al. 2026, J Ethnopharmacol 361:121250 | Published version of record | 10.1016/j.jep.2026.121250 |
| Li et al. 2026a, Chin Med 21:33 | Published version of record | 10.1186/s13020-025-01302-y |
| Li et al. 2026b, Mediators Inflamm 2026:7737168 | Published version of record | 10.1155/mi/7737168 |
| Shao et al. 2026, Chin J Nat Med 24:45-58 | Published version of record | 10.1016/S1875-5364(26)61076-X |
| Shen et al. 2026, J Inflamm Res 19:538988 | Published version of record | 10.2147/JIR.S538988 |
| Su et al. 2026, Phytomedicine 159:158554 | Published version of record | 10.1016/j.phymed.2026.158554 |
| Sun et al. 2026, Int J Biol Macromol 353:151310 | Published version of record | 10.1016/j.ijbiomac.2026.151310 |
| Xing et al. 2026, Int Immunopharmacol 175:116451 | Published version of record | 10.1016/j.intimp.2026.116451 |
| Yue et al. 2026, Food Sci Nutr 14:e71453 | Published version of record | 10.1002/fsn3.71453 |
| Zhang et al. 2026a, Int Immunopharmacol 174:116378 | Published version of record | 10.1016/j.intimp.2026.116378 |
| Zhang et al. 2026b, Fitoterapia 190:107157 | Published version of record | 10.1016/j.fitote.2026.107157 |
| Zhang et al. 2026c, J Chin Med Assoc 89:61-68 | 2026 issue; electronically published 19 June 2025 | 10.1097/JCMA.0000000000001259 |
| Zhang et al. 2026d, Front Immunol 17:1877804 | Published version of record | 10.3389/fimmu.2026.1877804 |
